# Intestinal parasitic infections and associated factors among street dwellers’ in Dessie town, North-East Ethiopia: a cross sectional study

**DOI:** 10.1186/s13104-019-4302-4

**Published:** 2019-05-10

**Authors:** Daniel Getacher Feleke, Edosa Kebede Wage, Tigist Getachew, Alemu Gedefie

**Affiliations:** Department of Medical Laboratory Science, College of Medicine and Health Sciences, Wollo University, Dessie, Ethiopia

**Keywords:** Intestinal parasites, Street dwellers, Ethiopia

## Abstract

**Objective:**

Intestinal parasitic infections are among the major cause of diseases of public health problems in sub-Saharan Africa. In Ethiopia, epidemiological information on street dwellers is very limited. So, this study aimed to determine the prevalence and associated factors of intestinal parasite among street dwellers’ in Dessie town, North-East, Ethiopia.

**Results:**

A cross-sectional study was carried out on street dwellers in Dessie town from November 2017 to February, 2018. Stool specimen was examined by direct wet mount, formol-ether concentration technique and modified Ziehl–Neelsen methods. Majority of study participants were males 220 (89.4%). The mean age of the study participants were 22.85 (SD = 4.78) years. The overall parasite prevalence was 108/246 (43.9%). Among the six different intestinal parasites detected, *H. nana* 33 (13.4) and *E. histolytica* 24 (9.8%) were dominant. Multivariate analysis showed, shoe wearing habit (P = 0.035), hand washing habit after toilet (P = 0.035), and history of animal contact (P = 0.016) had statistically significant association with intestinal parasitic infections after adjusting other variables. Although the prevalence of intestinal parasitic infections in this study was lower than previous studies conducted in similar study groups. The prevention and control strategies of intestinal parasites should address the poor segment of populations including street dwellers.

## Introduction

Intestinal parasitic infections are among major public health problems worldwide [1, 2]. It is estimated that about 3.5 billion people are affected, and that 450 million are ill as a result of these infections [3]. Intestinal parasitic infections are among the major public health problems in sub-Saharan Africa and cause morbidity and mortality [4]. They have been also associated with stunting, physical weakness and low educational performance of schoolchildren [5].

Intestinal parasitic infections are more prevalent among the poor segment of population. They are closely associated with low household income, poor personal and environmental sanitation, and overcrowding, limited access to clean water, tropical climate and low altitude [5, 6]. Street dwellers are among the most deprived people in urban areas, in terms of living conditions and lack of access to basic facilities and health indicators [7]. Access to health care for homeless individuals differs greatly from that for the general population. Street dwellers who visit health facilities may not get treatment due to financial problem and the morbidity is extremely high [7, 8].

Parasitic infections are widely distributed in Ethiopia due to low level of living standards, poor environmental sanitation and personal hygiene [2, 9]. Homeless people do not have access to safe water for drinking and for proper hygiene practice and lack of toilet facilities are the main contributors to the high prevalence of intestinal parasites in street dwellers. Due to lack of health service seeking behavior and treatment denial by health service providers, street dwellers can be a reservoir for intestinal parasites and make the prevention and control challenging. In Ethiopia, epidemiological information on the prevalence and associated factors of intestinal parasites in street dwellers is very limited. So, this study aimed to determine the prevalence and associated factors of intestinal parasite among street dwellers’ in Dessie town, North-East, Ethiopia.

## Main text

### Methods

#### Study area and study participants

This study was conducted from October, 2017 to January, 2018 on street dwellers in Dessie town, North-East, Ethiopia. Dessie town is located 401 km from Addis Ababa, the capital of Ethiopia. It is located at an altitude of 2470 m above sea level in low-shrouded mountains and hills” and the surrounding mountains. Based on the 2007 national census conducted by the Central Statistical Agency of Ethiopia (CSA), Dessie district has a total population of 151,174, of whom 72,932 are men and 78,242 women; 120,095 or 79.44% are urban inhabitants living in the town of Dessie. The number of street dwellers in Dessie town estimated to be more than 3000.

#### Study design

This cross-sectional study was conducted from October 2017 to January, 2018 on street dwellers in Dessie town, North-East Ethiopia.

#### Sampling technique and sample size determination

Street dwellers that fulfilled the inclusion criteria were selected by random sampling method.

Street dwellers whose age is above 2 years and who can able to provide stool specimen were included. Sample size was calculated using single proportion population formula.

$$n = \frac{{(Z_{a/2}^{2}) p\left( {1 - p} \right)}}{{d^{2} }}$$

It was calculated using a prevalence of 89.7% (10) with a margin of error 0.04 and a confidence level of 95%. In line with this, 246 study participants were recruited including the 10% non-response rate.

#### Ethical consideration

The study was conducted after obtaining ethical clearance from Wollo University ethical committee. A written consent form was used to ask the willingness of the study participants/or guardians. Intestinal parasite infected study participants were treated with the appropriate anti-parasitic drugs.

#### Data collection

The study sites were visited and data collectors were also trained before data collection. An interview based structured questionnaire was used to collect socio-demographic and other data from all street dwellers. The study participants were instructed how to bring the stool sample and were provided clean, dry leak proof labeled container with toilet paper.

#### Laboratory investigation

Stool specimen from each street dweller was examined by direct wet mount method using normal saline (0.85% NaCl solution) and Lugol’s iodine at the site of stool specimen collection. Stool samples were preserved with 10% formalin and formol-ether concentration technique was performed from each stool specimens. Samples were examined microscopically using the 10× and 40× objective lenses. Coccidian intestinal parasites were examined using modified Ziehl–Neelsen method.

#### Data analysis

Data quality was checked and were entered to SPSS version 20 software and analyzed. Logistic regression was done to investigate the relationship between the dependent and independent variables. P < 0.05 was considered statistically significant.

### Results

A total of 246 study participants were involved and majority of the study participants were males 220 (89.4%). The mean age of the study participants were 22.85 (SD = 4.78) years (age ranged from 15 to 36 years). Regarding the marital status, 242 (98.4%) of the study participants were single. Addiction associated problems and peer pressure were mentioned by the study participants as a reason for street dwelling with 86 (35.0%) and 84 (34.15%), respectively (Table 1).

**Table 1:** Socio demographic characteristics of the study participants

Variables	Category	Frequency	Percentage (%)
Sex	Female	26	10.6
	Male	220	89.4
Age (years)	< 20	95	38.6
	20–29	123	50.0
	> 29	28	11.4
Marital status	Single	242	98.4
	Married	1	0.4
	Widowed	1	0.4
	Divorced	2	0.8
Educational status	Illiterate	70	28.5
	Elementary	160	65.0
	High school	16	6.5
Enforcing factors for street dwelling	Poverty	48	19.5
	Addiction	86	35
	Divorce and family problem	12	4.9
	Pregnancy associated	4	1.6
	Peer pressure	84	34.15
	Education associated	12	4.934
Street dwelling duration	1 month	1	0.4
	1 year	81	32.9
	2 years	88	35.8
	> 2 years	76	30.9

#### Intestinal parasite prevalence in street dwellers

The overall parasite prevalence was 108/246 (43.90%). Six different intestinal parasites were detected. *H. nana* 33 (13.4) and *E. histolytica* 24 (9.8%) were the dominant helminthes and protozoan parasites, respectively. Taenia species 19 (7.7%) and *G. lamblia* 13 (5.3%) were also the most frequently detected parasites. The other two intestinal parasites detected were *A. lumbricoides* 11 (4.5%) and 8 (3.3%) *E. vermicularis*.

Single parasitic infection (35.8%) was prevalent followed by double infections (8.13%) and there were no triple infections. The prevalence of parasitic infection was higher in females. With regard to age of the study participants, intestinal parasitic infections were higher in children younger than 20 years (47.4%). Similarly, the prevalence of intestinal parasitic infection was higher in secondary school (56.25%) completed study participants (Table 2).

**Table 2:** The prevalence of intestinal parasitic infection among different socio-demographic characteristics

Characteristics	Number (%) of study participants	Number (%) positives for any parasite
Sex		
Male	220 (89.4)	95 (43.2)
Female	26 (10.6)	13 (50.0)
Age		
< 20	95 (38.6)	45 (47.4)
20–29	123 (50.0)	51 (41.5)
> 29	28 (11.4)	12 (42.9)
Marital status		
Single	242 (98.4)	108 (44.6)
Married	1 (0.4)	0 (0.00)
Widowed	1 (0.4)	0 (0.00)
Divorced	2 (0.8)	0 (0.00)
Educational status		
Illiterate	70 (28.5)	30 (42.85)
Primary school	160 (65.0)	69 (43.1)
Secondary school and above	16 (6.5)	9 (56.25)
Duration as a street dweller		
1 month	1 (38.6)	1 (100.0)
1 year	81 (50.0)	29 (35.8)
2 years	88 (11.4)	40 (45.5)
> 2 years	76 (0.0)	38 (50.0)

Intestinal parasites prevalence was higher among individuals who had not shoe wearing habit (61.53%) and among individuals who had frequent animal contact (55.4%). Similarly, intestinal parasitic infections were also higher in individuals who had not hand washing habit after toilet (45.3%). Univariate and multivariate logistic regression was done to investigate the relationship between dependant and independent variables. Goodness of fit of the model was checked using Hosmer and Lemeshow test (P > 0.05). Backward logistic regression method was used for multivariate analysis. In multivariate analysis, shoe wearing habit (P = 0.035), hand washing habit after toilet (P = 0.035), and history of animal contact (P = 0.016) had statistically significant association with intestinal parasitic infections after adjusting other variables. Multivariate logistic regression analysis was also showed that individuals who had animal contact are more likely to be infected by intestinal parasitic infection (AOR = 2.04, 95% CI 1.14–3.36) (Table 3).

**Table 3:** The prevalence of intestinal parasites in relation to associated risk factors

Risk factors	Intestinal parasite infection		COR (95% CI)	AOR (95% CI)	P-value
	Positive n (%)	Negative n (%)			
Sex					
Male	95 (43.4)	124 (56.6)	0.90 (0.4–2.04)		
Female	13 (48.1)	14 (51.9)	1		
Age					
< 20	45 (47.4)	51 (52.6)	0.99 (0.43–2.32)		
20–29	51 (41.5)	72 (58.5)	0.82 (0.36–1.87)		
> 29	12 (44.4)	15 (55.6)	1		
Educational status					
Illiterate	30 (42.9)	40 (57.1)	0.58 (0.20–1.74)		
Primary school	69 (43.1)	91 (56.9)	0.59 (0.21–1.66)		
Secondary school and above	9 (56.2)	7 (43.8)	*1*		
Shoe wearing habit					
Yes	84 (40.6)	123 (59.4)	2.43 (1.16–4.72)	0.46 (0.22–0.95)	*0.035*
No	24 (61.5)	15 (38.5)	1	1	
Buying from restaurants	2 (40.0)	3 (60.0)	0.86 (0.14–5.35)		
Food source					
Hotel	45 (44.1)	57 (55.9)	1.02 (0.61–1.73)		
Individual house	4 (50.0)	4 (50.0)	1.30 (0.31–5.42)		
Mixed (hotel, individual house and garbage)	58 (43.6)	75 (56.4)	1		
Type of latrine					
No latrine	89 (43.4)	116 (56.6)	0.77 (0.47–12.4)		
Public latrine	18 (46.2)	21 (53.8)	0.86 (0.05–14.7)		
Private latrine	1 (50.0)	1 (50)	1		
History of animal contact					
Yes	41 (55.4)	33 (44.6)	1.95 (1.12–3.38)	2.04 (1.14–3.36)	*0.016*
No	67 (39.0)	105 (61.0)	1	1	
Hand washing habit after animal contact					
Yes	5 (35.7)	9 (64.3)	0.69 (0.23–2.14)		
No	103 (44.4)	129 (55.6)	1		
Hand washing habit after toilet					
Yes	2 (16.7)	10 (83.3)	0.24 (0.05–1.13)	0.18 (0.04–089)	*0.035*
No	106 (45.3)	128 (54.7)	1	1	
Hand washing habit before meal					
Yes	8 (42.1)	11 (57.9)	0.92 (0.36–2.38)		
No	100 (44.1)	127 (55.9)	1		
Nail trimming habit					
Yes	15 (42.9)	20 (57.1)	0.95 (0.46–1.96)		
No	93 (44.1)	118 (55.9)	1		
Open defecation					
Yes	97 (44.1)	123 (55.9)	1.07 (0.47–2.45)		
No	11 (42.3)	15 (57.7)	1		

Italic values indicate significance of P-value (P < 0.05)

### Discussion

The prevalence of intestinal parasitic infection in street dwellers in the present study was 43.9%. This prevalence was lower than reports from Addis Ababa and Gondar city [1, 8]. This difference could be explained by variations in climatic condition of the study area, socio economic conditions, the methods employed for stool examination and the season of study. Dessie is a cold place which is located 2470 m above sea level. This condition is not considered as the most favorable climate for the existence of many of the intestinal parasites as a result, their occurrence might be reduced. The higher prevalence of intestinal parasites in females than males in the present study was consistent with the study conducted in Addis Ababa while it was not in agreement with a report from Jimma town studied on beggars [8, 10]. In this study six different intestinal parasites were detected and *H. nana* was the dominant intestinal parasites followed by *E. histolytica/dispar*, Taenia species and *G. lamblia*. The higher prevalence of *H. nana* and *E. histolytica/dispar* might be associated with improper fecal disposal and consumption of contaminated water, respectively. However, the high prevalence of *H. nana* and Taenia species in the present study was not in line with other studies conducted in Ethiopia and other countries [1, 8, 10]. The present study agreed with studies that reported geo-helminthes were dominant followed by the *E. histolytica/dispar* and *G. lamblia*. This indicates that lack of environmental sanitation and inadequate access of clean water are the main factors that expose street dwellers for intestinal parasitic infections and other communicable diseases. Taenia species was the third highest prevalent parasite next to *H. nana* and *E. histolytica/dispar* which was in agreement with a report from a study conducted among street dwellers in Addis Ababa [8]. This might be due to the consumption of unhygienic raw meat street dwellers get from slaughter houses. Compared to result reported from Sudanese street children, the prevalence of *G. lamblia* and *E. histolytica/dispar* in the present study was lower. On the other hand, the prevalence of *H. nana* in the present study was higher than Sudanese report. The higher prevalence of *H. nana* in the present study might be due to the reason that most of the street dwellers in the present study practiced open defection and the autoinfection characteristics of the parasite.

In this study the higher proportion of females were infected with intestinal parasite than males. This finding was in agreement with the study reported from street dwellers in Addis Ababa. Regarding age groups, the rate of intestinal parasitic was slightly higher in individuals younger than 20 years old. This might be due to the hygienic practice and frequent contact with contaminated soil while playing. This was in line with the report from Addis Ababa [8].

In the present study, there was statistically significant association between intestinal parasitic infection and animal contact, hand washing habit after toilet and shoe wearing habit.

In general, the prevalence of intestinal parasitic infections was still high in our study. Unless, prevention and control strategies of intestinal parasites addressed this segment of population, it is very challenging to prevent and control intestinal parasites.

## Limitations

In this study special diagnostic technique such as scotch tape *Entrobious vermicularis* and *Kato katz* for *Schistosoma mansoni* was not performed due to lack of resources.

## Data Availability

The authors confirm that all data underlying the findings are fully available without restriction. All relevant data are within the manuscript.
